# Genome-Wide Expression Profiling of Complex Regional Pain Syndrome

**DOI:** 10.1371/journal.pone.0079435

**Published:** 2013-11-14

**Authors:** Eun-Heui Jin, Enji Zhang, Youngkwon Ko, Woo Seog Sim, Dong Eon Moon, Keon Jung Yoon, Jang Hee Hong, Won Hyung Lee

**Affiliations:** 1 Research Institute for Medical Sciences, College of Medicine, Chungnam National University, Daejeon, Korea; 2 Department of Anesthesia and Pain Medicine, Chungnam National University Hospital, Daejeon, Korea; 3 Department of Anesthesiology and Pain Medicine, Samsung Medical Center, Sungkyunkwan University School of Medicine, Seoul, Korea; 4 Department of Anaesthesiology and Pain Medicine, College of Medicine, The Catholic University, Seoul, Korea; 5 Department of Anaesthesiology and Pain Medicine, College of Medicine, The Catholic University, Daejeon, Korea; 6 Department of Phamacology, College of Medicine, Chungnam National University, Daejeon, Korea; 7 Clinical Trials Center, Chungnam National University Hospital, Daejeon, Korea; Boston Children’s Hospital and Harvard Medical School, United States of America

## Abstract

Complex regional pain syndrome (CRPS) is a chronic, progressive, and devastating pain syndrome characterized by spontaneous pain, hyperalgesia, allodynia, altered skin temperature, and motor dysfunction. Although previous gene expression profiling studies have been conducted in animal pain models, there genome-wide expression profiling in the whole blood of CRPS patients has not been reported yet. Here, we successfully identified certain pain-related genes through genome-wide expression profiling in the blood from CRPS patients. We found that 80 genes were differentially expressed between 4 CRPS patients (2 CRPS I and 2 CRPS II) and 5 controls (cut-off value: 1.5-fold change and *p*<0.05). Most of those genes were associated with signal transduction, developmental processes, cell structure and motility, and immunity and defense. The expression levels of major histocompatibility complex class I A subtype (*HLA-A29.1*), matrix metalloproteinase 9 (*MMP9*), alanine aminopeptidase N (*ANPEP*), l-histidine decarboxylase (*HDC*), granulocyte colony-stimulating factor 3 receptor (*G-CSF3R*), and signal transducer and activator of transcription 3 (*STAT3*) genes selected from the microarray were confirmed in 24 CRPS patients and 18 controls by quantitative reverse transcription-polymerase chain reaction (qRT-PCR). We focused on the *MMP9* gene that, by qRT-PCR, showed a statistically significant difference in expression in CRPS patients compared to controls with the highest relative fold change (4.0±1.23 times and *p* = 1.4×10^−4^). The up-regulation of *MMP9* gene in the blood may be related to the pain progression in CRPS patients. Our findings, which offer a valuable contribution to the understanding of the differential gene expression in CRPS may help in the understanding of the pathophysiology of CRPS pain progression.

## Introduction

Complex regional pain syndrome (CRPS) is a chronic, progressive, and devastating pain syndrome that is characterized by spontaneous pain, hyperalgesia, allodynia, altered skin temperature, and motor dysfunction [1], [2]. CRPS is generally classified into 2 types by the absence or presence of nerve injury. Patients with CRPS type I show no nerve injury, while type II patients exhibit nerve injury [3].

Due to the phenotypic complexity of CRPS, it is difficult to conduct a human based genome-wide association study in CRPS. Nonetheless, microarray tools have been commonly used to identify novel biomarkers that are known to contribute to pain pathways in animal pain models. Genome-wide expression analyses have been successfully performed only in animals. A different regulation of 86 genes after nerve injury was detected by a cDNA microarray analysis of spinal nerves from a rat model of neuropathic pain [4]. Furthermore, 124 co-regulated genes were identified in 3 neuropathic pain models (spared nerve injury, chronic construction injury, and spinal nerve ligation) by gene expression profiling of the rat dorsal root ganglion (DRG). Additionally, following a microarray-based screening study in large international pain cohort [5], a genetic association study was performed using single nucleotide polymorphisms (SNPs) of the potassium channel alpha subunit, *KCNS1*.

In addition to animal studies, recent studies focused on the identification of novel molecules or genetic loci related to neuropathic pain in humans suffering from CPRS. A genetic association study conducted in CRPS patients and controls provided a new CRPS susceptibility locus (D6S1014) in human leukocyte antigen (*HLA*) class I region [6]. Uçeyler et al. compared the cytokine expression (at the mRNA and protein level) in the serum between CRPS II or CRPS I patients and controls. The mRNA and protein levels of transforming growth factor (TGF)-β1 and interleukin (IL)-2 were higher and those of IL-4 and IL-10 were lower in CRPS patients than in controls [7]. Furthermore, the levels of tumor necrosis factor (TNF) receptor and IL-1β in cerebrospinal fluid and serum were found to be related to pain intensity in CRPS II patients [8]. So far, there are no reports of a genome-wide expression profiling analysis successfully conducted in CRPS patients.

In this study, we analyzed the gene expression levels in the whole blood of CRPS patients using a genome-wide expression profiling analysis and identified the molecules that were highly expressed in CPRS depending on type-I or -II. These different transcriptional profiles in CPRS may contribute to the understanding of the pathogenesis of CRPS progression.

## Materials and Methods

### Ethics Statement

All individuals enrolled in this study provided written informed consent for blood collection and use. The study protocol was approved by the Institutional Review Board (IRB) of Chungnam National University Hospital, St. Mary’s Hospital, and Samsung Medical Center.

### Patients and Pain Evaluation

A clinical diagnosis of CRPS was established using the ‘Budapest criteria’ published by the International Association for the Study of Pain (IASP) 2007 [9]. CRPS I and CRPS II were distinguished by the presentation of nerve injury as defined by the IASP [10]. The diagnosis is CRPS I if there is no nerve lesion, while the diagnosis is CRPS II if a nerve lesion is present. Inclusion criteria included CRPS patients who received the medication of CRPS or CRPS related depressive disorder such as pregabalin (or gabapentin), tricycllic antidepressant, oipiods, acetaminophen, selective serotonin reuptake inhibitors, serotonin norepinephrine reuptake inhibitors, and benzodiazepine derivatives. Exclusion criteria included CRPS patients who received the medication described in the inclusion criteria as a cause of other neurologic disorders or any other medications not mentioned in the inclusion criteria (Table 1). Blood samples of CRPS patients obtained while taking their medications. The healthy control group was free of infectious diseases and pain disorders, and had undergone no recent surgery at the sampling time. Mechanoallodynia was determined by the pain evoked by the Von Frey hair or brush application. A pinprick or cold ice was to the lesion to detect hyperalgesia. Thermography has been used for the evaluation of temperature asymmetry (1 > C) or skin color change. The microarray analysis was conducted in 5 controls and 2 CRPS I and 2 CRPS II patients. We selected 2 CRPS I and 2 CRPS II samples considering allodynia, hyperalgesia, spontaneous pain, temperature change, vasomotor change, atrophic change symptoms, and a high RIN (RNA integrity number) value. The mean ages of the CRPS patients and controls were 46.6±10.1 and 44.7±4.5 y, respectively. CRPS patients (24) and 18 controls were used for quantitative real-time PCR (qRT-PCR) validation (Table 1).

**Table 1:** Characteristics of CRPS patients.

Patient/age (years)/gender	Diagnosis	Disease duration (years)	Location of symptoms	Allodynia	Hyperalgesia/spontaneous pain/temperature change	Vasomotor change (sudomotor)	Atrophic change (dystrophic)	Current medication	Array/qRT-PCR
P1/60/F	CRPS II	5.0	Left ankle	Yes	Yes	Yes	Yes	Pregabalin, nortriptyline, oxycodone, tramadol, codeine phosphate, topiramate, mirtazapine	qRT-PCR
P2/54/F	CRPS I	3.5	Right forearm	Yes	Yes	Yes	No	Pregabalin, nortriptyline, oxycodone, tramadol, acetaminophen, alprazolam, mirtazapine	qRT-PCR
P3/51/M	CRPS II	13.0	Right forearm	Yes	Yes	Yes	Yes	Pregabalin, nortriptyline, oxycodone, mirtazapine, venlafaxine, topiramate, alprazolam	Array/qRT-PCR
P4/46/M	CRPS II	2.8	Right lower leg	Yes	Yes	Yes	Yes	Pregabalin, nortriptyline, tramadol, acetaminophen	Array/qRT-PCR
P5/53/M	CRPS I	3.0	Left arm	Yes	Yes	Yes	Yes	Pregabalin, oxycodone, tramadol, acetaminophen, clonazepam, alprazolam	Array/qRT-PCR
P6/47/F	CRPS I	2.4	Both legs and arms	Yes	Yes	Yes	Yes	Pregabalin, nortriptyline, oxycodone, alprazolam	Array/qRT-PCR
P7/53/M	CRPS I	2.7	Right forearm	Yes	Yes	No	No	Pregabalin, nortriptyline, tramadol, acetaminophen	qRT-PCR
P8/21/M	CRPS I	1.5	Right ankle, foot and lower leg	Yes	Yes	Yes	Yes	Pregabalin, tramadol, acetaminophen	qRT-PCR
P9/41/M	CRPS I	3.7	Both legs	Yes	Yes	No	No	Pregabalin, nortriptyline, oxycodone, tramadol,escitalopram, duloxetine, clonazepam, trazodone,mirtazapine	qRT-PCR
P1/39/M	CRPS II	3.0	Left hand	Yes	Yes	Yes	Yes	Gabapentin, nortriptyline, oxycodone, mirtazapine	qRT-PCR
P11/43/M	CRPS II	5.0	Left upper extremity	Yes	Yes	Yes	Yes	Pregabalin, nortriptyline, oxycodone, tramadol, acetaminophen, trazodone, clonazepam, milnacipran, mirtazapine	qRT-PCR
P12/36/M	CRPS I	3.2	Right hand and lower arm	Yes	Yes	No	Yes	Gabapentin, hydromorphone, oxycodone, fentanyl patch, milnacipran, tianeptine, clonazepam	qRT-PCR
P13/41/M	CRPS II	1.5	Left knee and leg	Yes	Yes	Yes	Yes	Pregabalin, hydromorphone, IRcodon, acetaminophene, milnacipran, trazodone, escitalopram	qRT-PCR
P14/55/F	CRPS II	4.3	Left leg and foot	Yes	Yes	Yes	Yes	Pregabalin, nortriptyline, hydromorphone, fentanyl patch, IRcodon, duloxetine, milnacipran, alprazolam, trazodone	qRT-PCR
P15/44/M	CRPS II	5.5	Left face, right leg and arm	Yes	Yes	Yes	Yes	Pregabalin, nortriptyline, hydromorphone, IRcodon, acetaminophene, milnacipran	qRT-PCR
P16/52/F	CRPS I	5.0	Left lower leg	Yes	Yes	Yes	No	Pregabalin, nortriptyline, oxycodone,clonazepam, alprazolam	qRT-PCR
P17/60/M	CRPS I	1.7	Left forearm	Yes	Yes	Yes	No	Pregabalin, tramadol, acetaminophen, milnacipran	qRT-PCR
P18/57/F	CRPS I	11.0	Both legs and arms	No	Yes	No	No	Pregabalin, nortriptyline, alprazolam, zolpidem	qRT-PCR
P19/22/M	CRPS I	2.4	Light hand	Yes	Yes	No	No	Pregabalin, nortriptyline, tramadol	qRT-PCR
P20/45/M	CRPS II	4.1	Both legs	Yes	Yes	Yes	No	Pregabalin, nortriptyline, oxycodone, tramadol, codeine phosphate, mirtazapine, alprazolam	qRT-PCR
P21/55/M	CRPS II	6.0	Both legs and trunk	Yes	Yes	Yes	Yes	Pregabalin, nortriptyline, fentnyl patch, tramadol, IRcodon, clonazepam	qRT-PCR
P22/47/M	CRPS II	1.1	Right forearm	Yes	Yes	Yes	No	Pregabalin, nortriptyline, oxycodone, tramadol, acetaminophen	qRT-PCR
P23/44/M	CRPS I	5.5	Left shoulder	Yes	Yes	No	No	Gabapentin, nortriptyline, oxycodone, clonazepam	qRT-PCR
P24/53/M	CRPS I	1.2	Left arm	No	Yes	Yes	No	Pregabalin, nortriptyline, acetaminophen	qRT-PCR

CRPS, complex regional pain syndrome; F, female; M, male; qRT-PCR, quantitative real-time polymerase chain reaction.

### Genome-wide Transcriptional Profiling

Whole blood samples were collected using PAXgene blood RNA tubes (PreAnalytiX, Hilden, Germany). Total RNA was extracted using the TRIzol Reagent (Ambion, CA, USA) and purified using RNeasy columns (Qiagen, Valencia, USA) according to the manufacturer’s protocol. The concentration of the RNA was assessed using a NanoDrop spectrophotometer (NanoDrop Technologies, Inc., Wilmington, DE, USA). For quality control, RNA purity and integrity were evaluated by denaturing gel electrophoresis and the OD 260/280 ratio, and analyzed on an Agilent 2100 Bioanalyzer (Agilent Technologies, Palo Alto, USA).

For the genome-wide transcriptional profiling, 550 ng of total RNA was amplified, purified and labeled with biotin-NTP using an Illumina RNA amplification kit (Ambion, Austin, USA) according to the manufacturer’s instructions. Labeled cRNA (750ng) was hybridized to each Human HT-12 v.4 Expression BeadChip that contained 47,323 well- characterized transcripts for 16–18 h at 58°C, according to the manufacturer's instructions (Illumina, Inc., San Diego, USA). BeadChips were then washed and developed using Amersham fluorolink streptavidin-Cy3 (GE Healthcare Bio-Sciences, Little Chalfont, UK). Arrays were scanned with an Illumina BeadArray Reader confocal scanner. The microarray data are available at the Gene Expression Omnibus (GEO) website (http://www.ncbi.nih.gov/geo/; series GSE47603).

### Raw Data Processing and Statistical Analysis

The raw data were processed using the software provided by the manufacturer (Illumina GenomeStudio version 2011.1, Gene Expression Module v1.9.0. We applied a filtering criterion for data analysis: a high signal value was required to obtain a detection *p* value <0.05. The selected signal value of the probe was transformed using a logarithmic function and normalized using the quantile method. Statistical significance of the expression data was determined using independent t-test and fold change in which the null hypothesis was that no difference exists between the CRPS group and the control group. The false discovery rate (FDR <0.05) was controlled by adjusting the *p*- value using the Benjamini-Hochberg algorithm [11]. The data were further processed with 2 cut-off values, *p*-value <0.05 and fold change >1.5. A hierarchical cluster analysis was performed using complete linkage and Euclidean distance as a measure of similarity. The significant probe list was classified into biological process and molecular function using the panther classification system (http://www.pantherdb.org). All data analysis and visualization of differentially expressed genes (DEG) was conducted using R 2.14.1 (www.r-project.org).

### qRT-PCR Analysis

qRT-PCR was used to verify the differential expression that was initially detected by the array. Total RNA was isolated from whole blood using the TRIzol Reagent (Ambion, CA, USA). RNA concentration and purity were assessed using a NanoDrop spectrophotometer (NanoDrop Technologies, Inc., Wilmington, DE, USA). cDNA was synthesized using 1 µg of total RNA and QuantiTect Reverse Transcription Kit (Qiagen, CA, USA). PCR amplification was performed using cDNA, Power SYBR Green PCR Master Mix (Applied Biosystems, CA, USA), TaqMan Universal Master Mix II with UNG (Applied Biosystems, CA, USA), QuantiTect Primer Assay kit (Qiagen, CA, USA), and TaqMan Gene Expression Assay kit (Applied Biosystems, CA, USA); The genes assayed included *HLA-A29-1* (QT01341396), *HLA-DRB6* (QF00405783), *MMP9* (QT00040040), *PTGS2* (QT00040586), *IL-*8 (QT00000322), *G-CSF3R* (QT00095522), *ARHGEF10* (QT00196889), *GAPDH* (QT01192646), *HLA-DRB1* (Hs99999917_m1), *MMP2*5 (Hs01554789_m1), *ANPEP* (Hs00174265_m1), *HDC* (Hs00157914_m1), *STAT*3 (Hs00374280_m1) and *GAPDH* (Hs99999905_m1). Amplification reactions were performed in triplicate with a StepOne Plus system (Applied Biosystems, CA, USA) using the following conditions: 10 min at 95°C, 40 cycles of 15 s at 95°C and 1 min at 60°C for the primer assay; and 2 min at 50°C, 10 min at 95°C, 40 cycles of 15 s at 95°C and 1 min at 60°C for the probe assay. The threshold cycle (Ct) of the GAPDH gene was used as a reference control to normalize the expression level of the target gene (ΔCt) to correct for experimental variation. The relative level of gene expression (ΔΔCt) was calculated as ΔCt_CRPS patient_ − ΔCt_control_, and the relative fold changes were determined by using the 2^−ΔΔCt^ method [12]. Statistical analysis of the difference in gene expression (2^−ΔΔCt^ values) levels between CRPS patients and controls was calculated by a nonparametric Mann-Whitney *U* test (SPSS ver 20.0). A *p*-value <0.05 was considered to indicate statistical significance.

## Results

### Identification of DEGs in the Blood of CRPS

**Figure 1:**
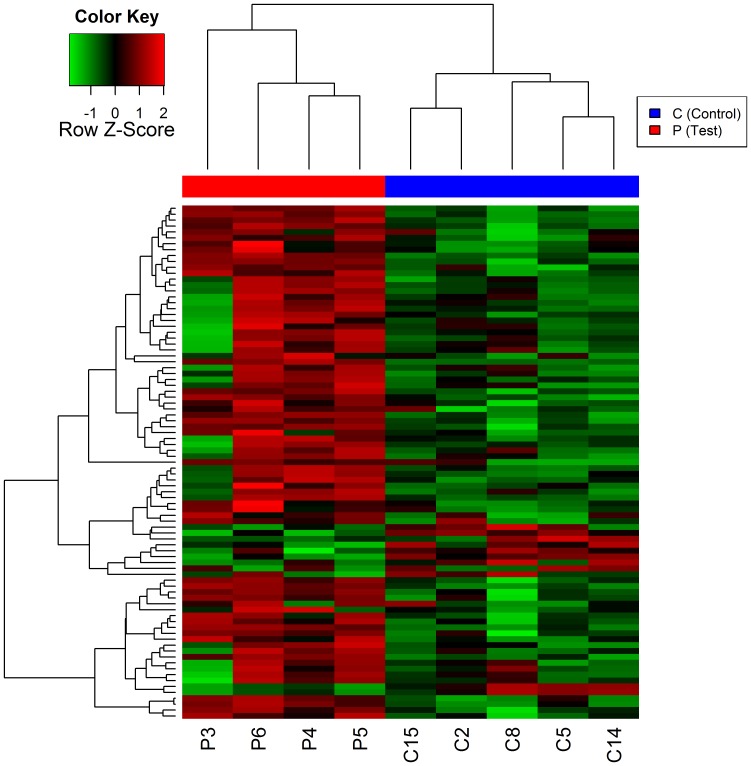
A heatmap based on gene expression patterns. Red and green represent an increase and the decrease in the gene expression levels, respectively, compared between 4 patients with complex regional pain syndrome (CRPS) and 5 controls. Fold change ≥1.5 and *p*<0.05.

**Figure 2:**
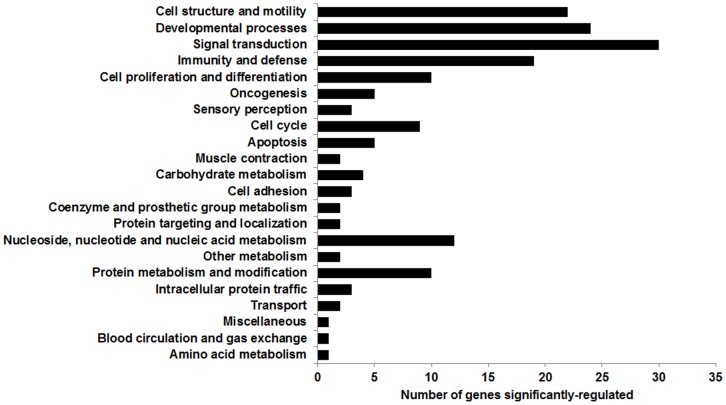
The functional categories of significantly regulated genes above 1.5-fold change (*p*<0.05). Each bar represents the percentage of up- and down-regulated genes in each category.

To identify DEGs between 4 CRPS patients and 5 controls, we performed a microarray analysis using the Human HT-12 v.4 Expression BeadChip. A heatmap analysis showed 80 DEGs with a 1.5-fold change cut-off values, *p*<0.05, and FDR <0.05 (Fig. 1). Among these, 69 genes were up-regulated and 11 genes were down-regulated (Table 2). The functional enrichment analysis for the 80 DEGs was performed by on the basis of the PANTHER classification system-based analyses (http://www.pantherdb.org) (Fig. 2). A classification of the genes according to their function revealed that they were associated with signal transduction, developmental process, cell structure and motility, and immunity and defense. Of the 80 DEGs, we selected the following 12 genes on the basis of a thorough literature review: HLA class II beta chain 1 (*HLA-DRB1*), *HLA-A29.1*, HLA class II beta chain 6 (*HLA-DRB6*) [13], *MMP9* [14], prostaglandin-endoperoxide synthase 2 (*PTGS2*) [15], *IL-8* [16], *MMP25* [17], alanine aminopeptidase N (*ANPEP*, or *CD13*) [18], l-histidine decarboxylase (*HDC*) [19], *G-CSF3R* [20], *STAT3* [21], and Rho guanine nucleotide exchange factor 10 (*ARHGEF10*) [22]. Compared to controls, *HLA-DRB1*, *HLA-A29.1*, *HLA-DRB6*, *MMP9*, *PTGS2*, *IL-8*, *MMP25*, *ANPEP*, *HDC*, *G-CSF3R*, and *STAT3* were up-regulated, while *ARHGEF10* was down-regulated in CRPS patients (Table 2).

**Table 2:** Eighty up- or down- regulated genes in CRPS patients.

Symbol	Gene name	Fold change	*p*-Value
HLA-DRB1	Major histocompatibility complex, class II, DR beta 1	14.9±0.2	<1×10^−29^
HLA-A29.1	Major histocompatibility complex, class I, A subtype	13.1±0.3	<1×10^−29^
CRISPLD2	Cysteine-rich secretory protein LCCL domain containing 2	3.1±0.1	6.1×10^−5^
HLA-DRB6	Major histocompatibility complex, class II, DR beta 6	3.1±0.8	8.0×10^−7^
MMP9	Matrix metallopeptidase 9	3.1±0.6	2.5×10^−7^
SNORD3D	Small nucleolar RNA, C/D box 3D	2.8±0.5	1.1×10^−9^
PTGS2	Prostaglandin-endoperoxide synthase 2	2.8±0.1	8.2×10^−5^
IL-8	Interleukin 8	2.7±0.4	1.1×10^−9^
MMP25	Matrix metallopeptidase 25	2.7±0.1	2.8×10^−5^
FOLR3	Folate receptor 3 (gamma)	2.6±0.8	0.006
ANPEP	Aminopeptidase N, or CD13	2.5±0.1	0.013
CMIP	c-Maf-inducing protein	2.5±0.1	2.6×10^−4^
POLR2A	POLR2A polymerase (RNA) II (DNA directed) polypeptide	2.4±0.03	0.003
ARID3A	ARID3A AT rich interactive domain 3A	2.4±0.1	0.002
HDC	L-histidine decarboxylase	2.4±0.1	0.003
LOC100130886	Hypothetical protein LOC100130886	2.3±0.1	0.002
PF4V1	Platelet factor 4 variant 1	2.3±0.5	3.0×10^−4^
DYSF	Dysferlin, limb girdle muscular dystrophy 2B	2.3±0.3	0.020
ACTN1	Actinin, alpha 1	2.3±0.1	0.004
ZYX	Zyxin	2.3±0.1	0.026
MYH9	Myosin, heavy chain 9, non-muscle	2.3±0.1	3.0×10^−4^
LOC730286	Hypothetical LOC730286	2.2±0.3	0.017
C15ORF39	Chromosome 15 open reading frame 39	2.2±0.1	0.025
G-CSF3R	Granulocyte colony stimulating factor 3 receptor	2.2±0.1	9.9×10^−5^
SLC25A24	Solute carrier family 25 (mitochondrial carrier; phosphate carrier), member 24	2.1±0.2	0.003
IL-17RA	Interleukin 17 receptor A	2.1±0.1	0.038
LOC100128326	Putative uncharacterized protein FLJ44672-like	2.1±0.3	0.030
TRIM58	Tripartite motif containing 58	2.1±0.6	1.4×10^−4^
SNORD3A	Small nucleolar RNA, C/D box 3A	2.1±0.7	4.4×10^−6^
ATHL1	Acid trehalase-like 1	2.0±0.1	0.046
LOC100134530	Hypothetical protein LOC100134530	2.0±0.3	0.046
WAS	Wiskott-Aldrich syndrome (eczema-thrombocytopenia)	2.0±0.1	0.007
STAT3	Signal transducer and activator of transcription 3	1.9±0.1	0.046
CARM1	Coactivator-associated arginine methyltransferase 1	1.9±0.4	0.001
NOD2	Nucleotide-binding oligomerization domain containing 2	1.9±0.2	0.046
RNU11	RNA, U11 small nuclear	1.9±0.4	0.003
RPRC1	MAP7 domain containing 1	1.9±0.1	0.024
LOC100132112	Similar to hCG1793472	1.9±0.3	0.031
EPB49	Erythrocyte membrane protein band 4.9	1.9±0.7	2.8×10^−5^
TMEM158	Transmembrane protein 158	1.9±0.7	0.001
FOXO3	Forkhead box O3	1.8±0.3	0.006
LOC100131164	Similar to anion exchanger	1.8±0.8	1.6×10^−6^
SPRYD5	TRIM51 tripartite motif-containing 51	1.8±0.1	0.004
BTG2	B cell translocation gene family, member 2	1.8±0.1	0.046
ZFP36	Zinc finger protein 36, C3H type, homolog	1.8±0.2	0.031
CA2	Carbonic anhydrase II	1.8±0.5	0.011
RNU1-5	RNA, U1 small nuclear 5	1.8±0.3	0.030
LOC100008588	RNA, 18S ribosomal 1	1.7±0.3	0.035
WDR40A	DDB1 and CUL4 associated factor 12	1.7±0.6	1.5×10–4
RBM38	RNA binding motif protein 38	1.7±0.5	2.6×10–4
C16ORF35	Chromosome 16 open reading frame 35	1.7±0.3	0.020
TREML3	Triggering receptor expressed on myeloid cells-like 3	1.7±0.2	0.046
NRGN	Neurogranin (protein kinase C substrate, RC3)	1.7±0.4	0.004
HS.562219	HS.562219	1.7±0.3	0.039
UBE2H	Ubiquitin-conjugating enzyme E2H	1.7±0.3	0.007
FOXO4	Forkhead box O4	1.7±0.2	0.015
ALAS2	Aminolevulinate, delta-, synthase 2	1.7±0.8	0.009
GMPR	Guanosine monophosphate reductase	1.7±0.6	0.004
BCL2L1	BCL2-like 1	1.7±0.7	2.2×10–4
IGF2BP2	Insulin-like growth factor 2 mRNA binding protein 2	1.6±0.4	0.021
E2F2	E2F transcription factor 2	1.6±0.5	0.002
RNU1-3	RNA, U1 small nuclear 3	1.6±0.3	0.041
JUNB	Jun B proto-oncogene	1.6±0.2	0.020
ADM	Adrenomedullin	1.6±0.4	0.025
RNF10	Ring finger protein 10	1.6±0.5	0.008
LOC440359	LOC440359	1.6±0.7	0.001
TUBB1	Tubulin, beta 1 class VI	1.5±0.3	0.035
ITGB2	Integrin, beta 2 (complement component 3 receptor 3 and 4 subunit)	1.5±0.1	0.006
FAM46C	Family with sequence similarity 46, member C	1.5±0.7	0.001
HLA-DQB1	Major histocompatibility complex, class II, DQ beta 1	-5.5±0.5	4.3×10–29
MYOM2	Myomesin (M-protein) 2	-3.4±1.0	5.6×10–17
LOC100133678	HLA class II histocompatibility antigen, DQ alpha 1 chain-like	-3.1±0.2	1.9×10–10
AMFR	Autocrine motility factor receptor, E3 ubiquitin protein ligase	-2.5±0.5	0.001
LOC642073	Similar to HLA class II histocompatibility antigen, DRB1-1 beta chain precursor	-2.5±0.4	8.7×10–7
CD47	CD47 molecule	-2.4±0.3	2.8×10–5
HLA-DQA1	Major histocompatibility complex, class II, DQ alpha 1	-2.2±0.2	2.8×10–5
ARHGEF10	Rho guanine nucleotide exchange factor (GEF) 10	-2.1±0.1	7.9×10–6
CD160	CD160 molecule	-2.0±0.2	0.021
CCL23	Chemokine (C-C motif) ligand 23	-1.8±0.2	0.046
RPL14	Ribosomal protein L14	-1.8±0.7	5.0×10–5

Using *t*-test with *p*-value <0.05 and false discovery rate <0.05. Fold changes are presented as mean ± SEM.

### Validation of DEGs in CRPS by qRT-PCR

**Figure 3:**
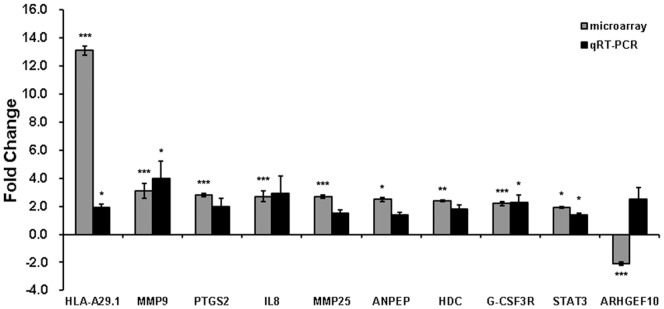
Validation of DEGs selected through microarray analysis by qRT-PCR. Bars represent the fold change in CRPS relative to controls for microarray data and qRT-PCR data and the fold change is the base-2 logarithm scale. Values are presented as mean ± standard error of the mean (SEM); n = 4 for microarray and n = 24 for qRT-PCR. Each experiment of qRT-PCR was performed in triplicate. Statistical significance is indicated by the number of star symbols; *p = 0.01 to <0.05; **p = 0.001 to 0.01; ***p<0.001.

**Figure 4:**
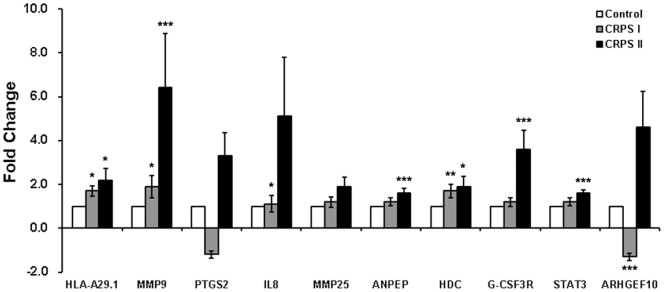
Quantitative qRT-PCR analyses of 10 selected genes. Bars represent the fold change, expressed as the mean ± standard error of the mean (SEM) in CRPS, CRPS I and CRPS II relative to controls. Fold changes were calculated relative to the average expression of controls by using the 2^∧^ (^−ΔΔCt^) method. Each experiment was performed in triplicate. Statistical significance is indicated by the number of star symbols; *p = 0.01 to <0.05; **p = 0.001 to 0.01; ***p<0.001.

To validate the DEGs selected from gene expression profiling, we performed qRT-PCR with 24 CRPS (13 CRPS I and 11 CRPS II) patients and 18 controls. The expression levels of *HLA-A29.1, MMP9, PTGS2*, *IL-8*, *MMP25*, *ANPEP*, *HDC*, *G-CSF3R*, and *STAT3* genes showed concordant results with the microarray data, while that of ARHGEF10 was not consistent with microarray results (Figure 3). The expression levels of 6 of those 10 genes (*HLA-A29.1*, *MMP9*, *ANPEP*, *HDC*, *G-CSF3R*, and *STAT3*) were significantly different between CRPS patients and controls (*p* = 0.004, 1.4×10^−4^, 0.017, 0.004, 0.017, and 0.017, respectively). The relative fold changes of *HLA-A29.1*, *MMP9*, *ANPEP*, *HDC*, *G-CSF3R*, and *STAT3* in the CRPS group compared to the control group were 1.9±0.26, 4.0±1.23, 1.4±0.14, 1.8±0.27, 2.3±0.48, and 1.4±0.12 times, respectively (Fig. 3). We also analyzed the gene expression levels in the subgroups CRPS I and CRPS II through a comparison of the 2^−ΔΔCt^ value between CRPS I or CRPS II patients and controls. We found that the expression level of *HLA-A29.1*, *MMP9*, *IL8*, *HDC*, and *ARHGEF10* showed a statistical difference between the CRPS I group and the control group (*p* = 0.011, 0.045, 0.045, 0.005, and 3.0×10^−4^, respectively). The relative fold changes of *HLA-A29.1*, *MMP9*, *IL8*, *HDC*, and *ARHGEF10* in the CRPS I group compared to the control were 1.7±0.23, 1.9±0.51, 1.1±0.38, 1.7±0.31, and -1.3±0.17 (Fig. 4). We also observed that the expression level of *HLA-A29.1*, *MMP9*, *ANPEP*, *HDC*, *G-CSF3R*, and *STAT3* significantly differed in the CRPS II patients, when compared to that in the controls (*p* = 0.020, 3.4×10^−7^, 3.0×10^−5^, 0.020, 3.0×10^−5^, and 3.0×10^−5^, respectively). There was a 2.2±0.51, 6.4±2.47, 1.6±0.22, 1.9±0.48, 3.6±0.89, and 1.6±0.16-fold increase in the expression of *HLA-A29.1*, *MMP9*, *ANPEP*, *HDC*, *G-CSF3R*, and *STAT3*, respectively, in the CRPS-II group compared to the control (Fig. 4).

## Discussion

Although the pathophysiology of neuropathic pain has not been completely understood, previous studies implicate that trauma induces activation of mast cells and macrophages and neutrophils are recruited to the injury region [23], [24]. Studies have been demonstrated that CRPS are associated with inflammatory and neuroinflammatory mediators in blood of patients compared to controls [25], [26]. Based on previous reports, we investigated the gene expression profiling in the blood of CRPS patients. In the present study, by genome-wide expression profiling followed by qRT-PCR validation, we found that *HLA-A29.1*, *MMP9*, *ANPEP*, *HDC*, *G-CSF3R*, and *STAT3* genes were highly expressed in the blood of CRPS patients, compared to controls (Fig. 3). Though pain-related genes have been identified through microarray analyses in animals, no reports of successful genome-wide transcriptional profiling in CRPS have been published. This is the first successful genome-wide expression profiling analysis in the blood of CRPS patients.

We observed fold change in *HLA-DRB1* and *HLA-DRB6* expression were the largest among the 80 genes that were up- or down-regulated in the microarray (14.9-fold and 3.1-fold, respectively) (Table 2). However, when examined by qRT-PCR, we were not able to confirm this microarray finding. Additionally, the expression level of *ARHGEF10* in qRT-PCR was inconsistent with that in microarray (Fig. 3).

In our subgroup analysis, the expression level of *HLA-A29.1*, *MMP9*, *ANPEP*, *HDC*, *G-CSF3R*, and *STAT3* genes in both CRPS group and CRPS II subgroup was statistically different (as assessed by the 2^−ΔΔCt^ value) compared to that of the control group. Fold changes in the expression of *HLA-A29.1*, *MMP9*, *ANPEP*, *HDC*, *G-CSF3R*, and *STAT3* genes in the CRPS II subgroup (2.2±0.51, 6.4±2.47, 1.6±0.22, 1.9±0.48, 3.6±0.89, and 1.6±0.16 times, respectively) were higher than for the CRPS (1.9±0.26, 4.0±1.23, 1.4±0.14, 1.8±0.27, 2.3±0.48, and 1.4±0.12 times, respectively) compared to the control. The expression level of *HLA-A29.1*, *MMP9*, *IL8*, *HDC*, *G-CSF3R*, *STAT3* and *ARHGEF10* showed statistical difference in CRPS I subgroup compared to that of the control group (Fig. 4). There are literature evidences on the involvements of *HLA-A29.1*, *MMP9*, *IL8*, *ANPEP*, *HDC*, *G-CSF3R*, *STAT3,* and *ARHGEF10* genes in pain progression. A HLA polymorphism was associated with postherpetic neuralgia in a Japanese population [13]. *MMP9* was up-regulated in dorsal root ganglion neurons of spinal nerve-ligated rats [23]. IL-8, a proinflammatory cytokine, induced hyperalgesia in rats [16]. In peripheral inflamed tissues of injured rats, pain was reduced by inhibition of opioid degradation with ANPEP or neutral endopeptidase [18]. HDC is the enzyme that produces histamine and participates in central pain modulation; intrathecal administration of histamine evoked hyperalgesia in *HDC* knockout mice [19]. The expression of *G-CSF* increased in a mouse model of bone tumor-induced pain and G-CSF signaling via its receptor led to nerve remodeling and bone cancer pain [20]. STAT3 has been shown to play an important role in inducing astrocyte proliferation and tactile allodynia in a neuropathic pain rat model [21]. ARHGEF10 was found to play an important role in myelination of peripheral nerves [22].

Based on previous studies and our results, we could assume that the direction of regulation of *HLA-A29.1*, *MMP9*, and *HDC* genes may be the same in both CRPS I and CRPS II, although the level of regulation in CRPS II was greater than that of CRPS I, that the up-regulation of *IL8* and the down-regulation of *ARHGEF10* gene may be related with the pain progression of CRPS I, and that the up-regulation of *ANPEP*, *G-CSF3R*, and *STAT3* genes may be associated with the pathogenesis of CRPS II. Interestingly, the expression of *MMP9* of validated genes was prominently up-regulated in subgroups CRPS I (1.9±0.26 times and *p* = 0.045) and CRPS II patients (6.4±2.47 times and *p* = 3.4×10^−7^) (Fig. 4). Thus, we particularly focused on the MMP9 gene expression.

There has been interesting evidence that supports the involvement of MMP9 in neuropathic pain. Matrix metalloproteinases are a family of endopeptidases that play an important role in neuroinflammation, developmental processes, and wound healing [27], [28]. MMP9 is one of the major gelatinases. MMP9 was up-regulated in rat DRG after a sciatic nerve crush that led to demyelination and its levels were regulated by TNF-α and IL-1β [29]. *MMP9* was also up-regulated in the DRG neurons of spinal nerve-ligated rats and induced neuropathic pain by cleaving IL-1β in the dorsal root ganglion and spinal cord; *MMP9*-null mice showed a reduction of pain in the form of mechanical allodynia [30]. Elevated MMP9 levels were observed in the plasma of migraineurs, even during headache- free periods. [14].

There are some limitations to our study. First, the sample size was too small to have statistical power. Second, all CRPS patients who participated in this study took several pain medications, such as pregabalin, gabapentin, tricyclic antidepressants, and opioids. Thus, we cannot rule out that the medications had an effect on the gene expression. To adequately control for this possibility further studies would be required with a control group of medication only. Third, CRPS patients that participated in this study were heterogenous with respect to disease duration.

In conclusion, based on the genome-wide gene expression profiling in the blood of CRPS patients, we suggest that the up-regulation of the *MMP9* gene in the blood might be related to pain progression in CRPS, although further replication and functional studies conducted in large populations are required to define the role of this gene in CRPS. This study offers an early and fascinating assay of gene expression in peripheral leukocytes in CRPS patients, one which may lead to new mechanisms and therefore potentially new therapies.
